# Predictors of Mortality in Bicycle-Related Trauma: An Eight-Year Experience in a Level One Trauma Center

**DOI:** 10.3390/jpm12111936

**Published:** 2022-11-21

**Authors:** Elisa Reitano, Stefano Piero Bernardo Cioffi, Francesco Virdis, Michele Altomare, Andrea Spota, Osvaldo Chiara, Stefania Cimbanassi

**Affiliations:** 1 Division of General Surgery, Department of Translational Medicine, Maggiore della Carità Hospital, University of Eastern Piedmont, Corso Giuseppe Mazzini 18, 28100 Novara, Italy; 2 General Surgery and Trauma Team, ASST Niguarda, Piazza Ospedale Maggiore 3, 20162 Milan, Italy; 3 Department of Medical-Surgical Physiopathology and Transplantation, University of Milan, Festa del Perdono 7, 20122 Milan, Italy

**Keywords:** emergency surgery, trauma center, trauma, emergency medicine

## Abstract

Background: Bicycle-related trauma has increased during the last decades, mainly due to the antipollution environmental policies. This study investigates the outcome of bicycle-related trauma in our level-one trauma center over a period of eight years. Methods: Data from 446 consecutive bicycle-related trauma patients admitted to our trauma center from 2011 to 2019 were selected and retrospectively analyzed. The sample was divided into three age groups: <18 years, 18–54 years, and ≥55 years. Mortality rates were obtained for the overall population and patients with an Injury Severity Score (ISS) ≥ 25. Month and seasonal patients’ distribution was described to provide an epidemiological overview of bike-related trauma over the years. Results: Patients ≥ 55 years showed a lower pre-hospital and in-hospital GCS (*p* ≤ 0.001), higher levels of lactates (*p* < 0.019) and higher ISS (*p* ≤ 0.001), probability of death (*p* ≤ 0.001), and overall mortality (*p* ≤ 0.001). The head and chest Abbreviated Injury Scale (AIS) ≥ 3 injuries were predictors of mortality, especially in patients over 55 years (*p* < 0.010). Bicycle-related trauma was more frequent during the summer (34%), particularly in July and August. Conclusions: Age over 55 years old, head and chest injuries, and an ISS > 25 were independent predictors of mortality.

## 1. Introduction 

In recent years, cycling has become more popular than in the past for transport and for sports purposes [1]. Bicycle-related trauma is widespread in urban areas, representing a consistent percentage of road traffic victims worldwide (2) [2]. Despite the injury prevention policies promoting helmet use and changes in bicycle infrastructure over the years, bicycle-related trauma seems to be even more frequent [3,4]. The spread of bicycle use was encouraged by the environmental antipollution policies and the diffusion of ride-sharing companies [3]. Unlike the other road traffic trauma, cyclists are more inclined to break road rules. This is also related to the geographic area (rural/urban) and to the availability of cycle tracks. Moreover, helmet use is not mandatory depending on the country’s regulation, and it is not even provided by most sharing companies [5,6].

Most of the studies on bicycle-related trauma investigated the epidemiological and social impact of helmet use on cyclists, often with conflicting results [7,8].

This study, performed in a level-one trauma center, aims to investigate the outcome of bicycle-related trauma according to age, site of injuries, and seasonal distribution to improve the level of care for this type of patient. 

## 2. Material and Methods

All patients consecutively admitted to Niguarda hospital, a level-one trauma center in Milan, Italy, for bicycle-related trauma from October 2011 to October 2019 were retrospectively retrieved from our Trauma Registry. 

All patients registered in our Trauma Registry following bicycle accidents with complete data were included in the study. The registry is held by a Trauma Team consultant who is meant to keep it constantly updated, and it is annually revised by the head of the department.

The study was conducted according to the principles declared by the National Commission for Data Protection and Liberties (CNIL: 2210699) and the ethical principles described in the Declaration of Helsinki. Our Trauma Registry project was approved by the local IRB: 534-102018. 

Demographic data, the mechanism of trauma, pre-hospital and in-hospital vital signs, trauma aggravating factors, the abbreviated injury scale (AIS) of each anatomical region, the Injury Severity Score (ISS), and probability of death (PD) estimated by the Trauma and Injury Severity Score (TRISS) system were considered. The American Society of Anesthesiologists’ (ASA) clinical status summarized patients’ comorbidities. Injuries were grouped by anatomical region according to AIS classification: head, face, chest, abdomen, extremities, and external. Patients were divided into three age groups to describe their injuries and mortality distribution (<18 years, 18–54 years, and ≥55 years), while an ISS equal or higher than 25 was chosen as the severity cut-off. Age and ISS cut-off were chosen accordingly to our previous study [9] on predictors of mortality among two-wheeled vehicles. 

Data were recorded in a computerized spreadsheet (Microsoft Excel 2016; Microsoft Corporation, Redmond, WA, USA) and analyzed with statistical software (IBM Corp., released 2012, IBM SPSS Statistics for Windows, Version 21.0; Armonk, NY, USA). The sample distribution was evaluated with the Kolmogorov–Smirnov and Shapiro–Wilk tests resulting in a non-Gaussian distribution for any examined variable. Continuous variables were compared by an independent sample Kruskal–Wallis test, while categorical variables were analyzed using Pearson’s chi-squared test. Two logistic regression analyses identified the association between AIS score and mortality and between aggravating factors and mortality by age groups, estimating the adjusted odds ratio (OR) and 95% confidence interval (CI). Variables with a *p*-value < 0.05 at the univariate analysis were included in the model. Survival curves were obtained with Kaplan–Meier analysis, and the log-rank test was assessed to evaluate differences in cumulative survival among age groups. Mortality rates were obtained for the overall population and patients with ISS ≥ 25, considering the age group stratification. Finally, monthly and seasonal patients’ distribution was described to provide an epidemiological overview of bike-related trauma over the year. 

## 3. Results

During the study period, four hundred and forty-six bicycle-related trauma patients were managed at our center. A total of 75 (16.8%) patients were younger than 18 years, 223 (50%) were between 18 and 54 years old, and 148 (33.2%) were older than 55 years. Patients ≥ 55 years showed lower pre-hospital and in-hospital GCS (*p* ≤ 0.001), higher levels of lactates (*p* < 0.019), ISS (*p* ≤ 0.001), probability of death (*p* ≤ 0.001) and overall mortality (*p* ≤ 0.001), as shown in Table 1. 

AIS < 3 head, face, chest, abdomen, and extremity minor injuries were more frequent in patients between 18 and 54 years, while AIS ≥ 3 head, chest, abdomen, and extremity injuries were more frequent in patients over 55 years.

Table 2 shows the mortality distribution for age groups according to AIS grading for each body region. The logistic regression model showed that AIS ≥ 3 head and chest injuries were predictors of mortality in patients over 55 years.

Table 3 shows mortality distribution for age groups following aggravating factors. Roll on and roll over was related to higher mortality in patients over 55 years, while thrown was an independent predictor of mortality between 18 and 54 years. Not wearing a helmet was not an independent predictor of mortality in all age groups.

**Figure 1:**
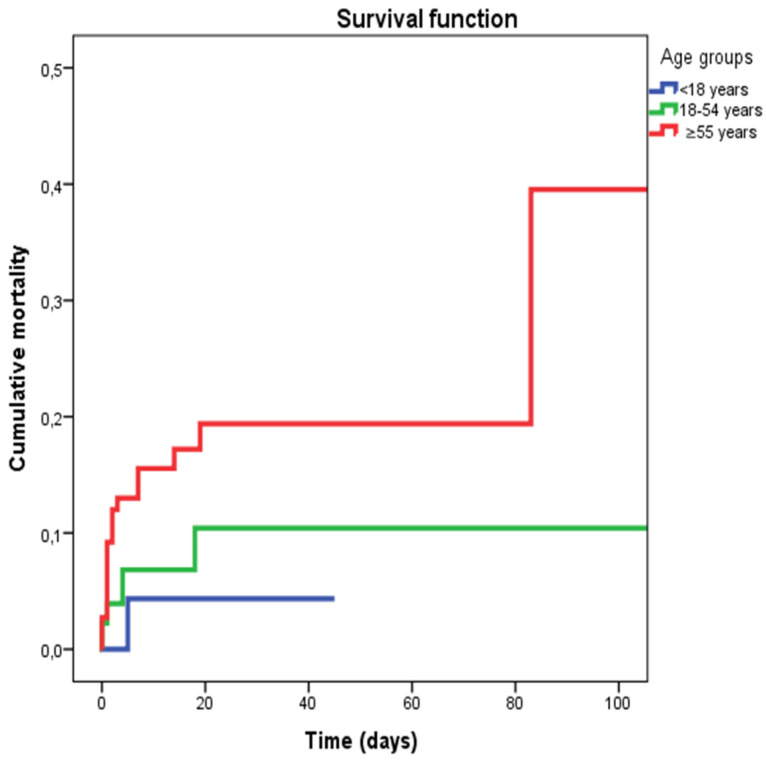
Survival function among the three age groups.

A total of 32 patients (7.2%) died: 6 patients (1.34%) with an ISS < 24 and 26 patients (26.5%) with an ISS ≥ 25 (log-rank test ≤ 0.001). The survival rates were 98.3% in patients with an ISS < 24 and 73.5% in patients with an ISS ≥ 25, respectively. Figure 1 shows the global mortality distribution among the age groups.

Patients over 55 years showed a worse prognosis between the age groups, with a survival rate of 86.4%. The survival rate of patients < 18 years was 98.7%, while between 18 and 54 years, the survival rate was 95.1% (log-rank test ≤ 0.028). Mortality distribution in patients with an ISS ≥ 25 showed higher mortality in patients over 55 years; although not statistically significant (log-rank test ≤ 0.701). 

**Figure 2:**
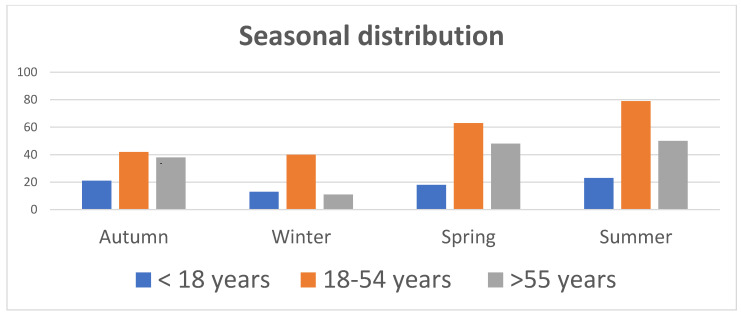
Seasonal distribution among the three age groups.

**Figure 3:**
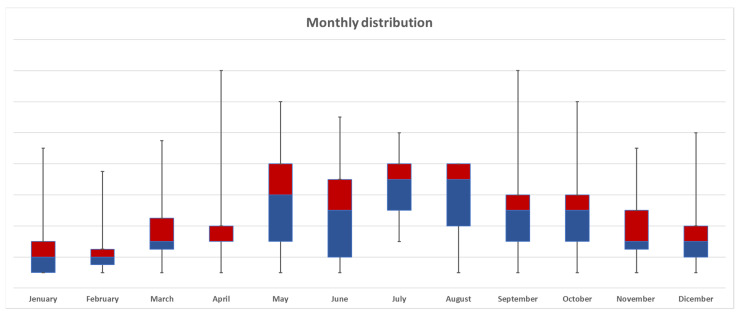
Overall monthly distribution of bike-related trauma, median and IQR graphic representation. IQR: interquartile range.

Bicycle-related trauma was more frequent in summer (34%), followed by spring (28.9%), autumn (22.6%), and winter (14.3%). Seasonal trauma distribution among the age groups is described in Figure 2, while monthly distribution is in Figure 3, with July and August more represented. 

## 4. Discussion 

Bicycle-related trauma represents an essential percentage of road traffic victims worldwide [2,10]. The increasing diffusion of this type of transport responds to antipollution policies, representing a valid alternative method of mobility in heavy-traffic urban areas [8,11]. Bicycle mobility was also improved by the diffusion of different bike-sharing companies worldwide, which has made this popular type of transport easily available in a few minutes [12]. Given the high diffusion of bicycle use both for sports and transport purposes, analyzing the kind of injuries and the mortality distribution of these types of trauma is of paramount importance. 

Our study confirmed head and chest injuries as independent predictors of mortality only in patients over 55 years old, as shown in Table 2. Aggravating factors influenced the mortality trends, as roll on and roll over were independent predictors of mortality in patients ≥ 55 years old. Thrown was an independent predictor of mortality between 18 and 54 years old (Table 3). Only 44 patients (9.86%) were not wearing helmets in our study; only one patient died among them, in the group 18–54 years old. In our research, not wearing a helmet was not an independent predictor of mortality, but head and chest AIS ≥ 3 were independent predictors of death. Therefore, despite the protective effect of the helmet, other variables should influence the mortality trends in cycle trauma. These results align with the study of Foley J. et al. [3], who showed that different variables (i.e., gender and mechanism of trauma) were independent predictors of mortality in bicycle trauma, and factors other than wearing a helmet could have a role in head injuries. A systematic review conducted by Hoye A. [13] showed that mandatory bicycle helmet legislation for all cyclists reduces about 20% of head injuries, significantly affecting severe head injuries. Two meta-analyses [14,15] confirmed the positive impact of the helmet only on severe head injuries, also showing a protective result on fatal injury prevention. However, all the studies agreed [3,15] on the role of different variables on bicycle mortality. 

The survival rate estimated with the Kaplan–Mayer method showed a higher mortality in patients with an ISS ≥ 25, confirming that overall trauma severity influenced mortality. 

However, as shown in Figure 1, patients older than 55 years showed the lowest survival rate, confirming age as an independent predictor of mortality. Interestingly, another study on motorcycle-related trauma [9] showed similar results, with a higher mortality trend in older patients (≥55 years old). 

Finally, Figure 2 shows the seasonal distribution of bicycle trauma: more frequent in summer (34%), followed by spring (28.9%). These results align with the current literature [16,17]. 

Given its retrospective nature, this study presents some limitations. Although older patients showed a higher mortality rate, it is possible that patients’ comorbidities and the use of anticoagulant therapy could influence the prognosis. Unfortunately, these variables were not available in our Trauma Registry and were not considered. 

Moreover, although this study was conducted in a level-one trauma center in Italy, no information on pre-hospital trauma mortality was available. Indeed, our data referred only to our in-hospital experience.

Finally, despite different studies showing a possible correlation between the bicycle infrastructure and mortality [18,19], this variable is not reported in our Trauma Registry, and it has not been considered. 

## 5. Conclusions

This study showed that different variables influenced bicycle trauma mortality. Older age and aggravating factors are independent predictors of mortality. Despite the protective effect of the helmet, head and chest injuries were confirmed to be independent predictors of mortality in patients ≥ 55 years old. Bicycle-related trauma is more frequent during the warm seasons, especially in July and August. Further multicentric and prospective studies should be advisable to confirm our results, fostering a stronger scientific collaboration with the pre-hospital care services.

## Figures and Tables

**Table 1:** Demographic and trauma-related data.

Variables	<18 Years 75 (16.8)	18–54 Years 223 (50)	≥55 Years 148 (33.2)	*p* Value
Male n (%)	58 (77.3)	167 (74.6)	120 (81.6)	0.281
GCS (median (IQR))	15 (15–15)	15 (14–15)	15 (13–15)	0.001 *
GCS Hospital (median (IQR))	15 (15–15)	15 (15–15)	15 (10–15)	0.001 *
DBP (median (IQR))	75 (60–80)	77 (70–80)	80 (70–94)	<0.001
SBP (median (IQR))	120 (112–130)	130 (120–140)	146 (125–165)	<0.001
HR (median (IQR))	100 (84–115)	80 (70–96)	80 (70–94)	≤0.001 *
RR (median (IQR))	18 (14–18)	16 (15–18)	16 (12–18)	0.006 *
BE (median (IQR))	−2 (−3.45–0.25)	−1.05 (−3.52–0.90)	−1.4 (−3.75–0.30)	0.499
Lactate (median (IQR))	1.82 (1.36–2.35)	1.79 (1.30–2.60)	2.11 (1.49–2.97)	0.019 *
OAC (median (IQR))	1.12 (1.06–1.16)	1.05 (1–1.12)	1.06 (1–1.12)	≤0.001 *
No Helmet n (%)	6 (8.1)	24 (10.7)	14 (9.5)	0.706
Roll on n (%)	2 (2.6)	7 (3.1)	6 (4.1)	0.825
Roll over n (%)	34 (45.3)	114 (50.9)	69 (46.9)	0.621
Throw n (%)	16 (21.3)	29 (12.9)	13 (8.8)	0.033 *
Fall without accident n (%)	25 (33.3)	70 (31.3)	49(33.3)	0.895
Cardioaspirin n (%)	0	0	14 (9.5)	≤0.001 *
OAC n (%)	0	0	3 (2.0)	0.046 *
ASA SCORE n (%)				≤0.001 *
I	74 (98.7)	196 (87.5)	48 (32.7)	
II	1 (1.3)	26 (11.6)	80 (54.4)	
III	0	2 (0.9)	19 (2.9)	
Emergency surgery n (%)	14 (18.7)	48 (21.4)	35 (23.8)	0.671
Elective surgery n (%)	6 (8)	39 (17.4)	36 (24.5)	0.010 *
Head AIS ≥ 3 n (%)	10 (2.2)	57 (12.8)	70 (15.7)	≤0.001 *
Face AIS ≥ 3 n (%)	0	3 (0.7)	3 (0.7)	0.460
Chest AIS ≥ 3 n (%)	10 (2.2)	52 (11.7)	47 (10.5)	0.008 *
Abdomen AIS ≥ 3 n (%)	8 (1.8)	10 (2.2)	5 (1.1)	0.055
Extremity AIS ≥ 3 n (%)	4 (0.9)	21 (4.7)	24 (5.4)	0.026 *
Dead n (%)	1 (1.3)	11 (4.9)	20 (13.6)	≤0.001 *
Length of stay (median (IQR))	3 (0–12)	1 (0–9.75)	5 (0–19)	≤0.001 *
ISS (median (IQR))	5 (2–14)	6 (2–17)	17 (6–29)	≤0.001 *
Probability of death (median (IQR))	0.40 (0.3–1)	0.50 (0.30–1.60)	6.10 (2.5–16.5)	≤0.001 *

GCS: Glasgow Coma Scale; DBP: diastolic blood pressure; SBP: systolic blood pressure; HR: heart rate; RR: respiratory rate; BE: base excess; INR: international normalized ratio; OAC: oral anticoagulation; AIS: Abbreviated Injury Scale; ISS: Injury Severity Score; IQR: interquartile range; * statistical significance.

**Table 2:** AIS distribution in age groups.

	Univariate Analysis			Multivariate Analysis	
	AIS < 3 n (%)	AIS ≥ 3 n (%)	*p* Value	OR (95%CI)	*p* Value
<18 years old					
Head	0	1 (1.3)	0.010 *	0	0.997
Abdomen	1 (1.3)	0	0.728		
Face	0	0	0		
Chest	0	1	0.010 *	0	0.997
Extremity	1 (1.3)	0	0.811		
External	1 (1.3)	-	-		
18–54 years old					
Head	0	11 (4.9)	≤0.001 *	0	0.995
Abdomen	10 (4.5)	1 (0.4)	0.446		
Face	10 (4.5)	1 (0.4)	0.022 *		
Chest	5 (2.2)	6 (2.7)	0.012 *	0.70 (0.18–2.62)	0.597
Extremity	10 (4.5)	1 (0.4)	0.974		
External	11 (4.9)	-	-		
≥55 years old					
Head	4 (2.7)	16 (10.9)	0.002 *	0.186 (0.05–0.61)	0.005 *
Abdomen	20 (13.6)	0	0.367		
Face	20 (13.6)	0	0.487		
Chest	7 (4.8)	13 (8.8)	≤0.001 *	0.198 (0.07–0.56)	0.002 *
Extremity	14 (9.5)	6 (4.1)	0.075 *		
External	20 (13.6)	-	-		

* Statistical significance; AIS: Abbreviated Injury Scale; OR: odds ratio; CI: confidence interval.

**Table 3:** Aggravating factors according to outcome.

	Univariate Analysis			Multivariate Analysis	
Aggravating Factors	Survived n (%)	Dead n (%)	*p* Value	OR (95%CI)	*p* Value
<18 years old					
No helmet	6 (8.0)	0	0.76		
Roll on	2 (2.7)	0	0.86		
Roll over	15 (20)	1 (1.3)	0.05		
Thrown	33 (44)	1 (1.3)	0.26		
18–54					
No helmet	24 (10.7)	1 (0.4)	0.82		
Roll on	6 (2.7)	1 (0.4)	0.22		
Roll over	25 (11.2)	4 (1.8)	0.01 *	0.30 (0.08–1.15)	0.079
Thrown	104 (46.8)	10 (4.5)	0.006 *	0.10 (0.01–0.87)	0.037 *
≥55 years old					
No helmet	14 (9.5)	0	0.11		
Roll on	3 (2)	3 (2)	0.008 *	0.11 (0.02–0.76)	0.012 *
Roll over	9 (6.1)	4 (2.7)	0.05 *	0.25 (0.06–0.94)	0.041 *
Thrown	6 (42.2)	7 (4.8)	0.25		

OR: odds ratio; CI: confidence interval; * statistical significance.

## Data Availability

The data presented in this study are available on request from the corresponding author. The data are not publicly available to preserve confidentiality.
